# Cancer Therapy by Catechins Involves Redox Cycling of Copper Ions and Generation of Reactive Oxygen Species

**DOI:** 10.3390/toxins8020037

**Published:** 2016-02-04

**Authors:** Mohd Farhan, Husain Yar Khan, Mohammad Oves, Ahmed Al-Harrasi, Nida Rehmani, Hussain Arif, Sheikh Mumtaz Hadi, Aamir Ahmad

**Affiliations:** 1 Department of Biochemistry, Faculty of Life Sciences, AMU, Aligarh 202002, India; farhan@mohdfarhan.com (M.F.); nida.rehmani4@gmail.com (N.R.); arifkap@gmail.com (H.A.); 2 UoN Chair of Oman’s Medicinal Plants and Marine Natural Products, University of Nizwa, Birkat Al Mauz, PO Box 33, Postal Code 616, Nizwa, Oman; husainyar@gmail.com (H.Y.K.); aharrasi@unizwa.edu.om (A.A.-H.); 3 Center of Excellence in Environmental Studies, King Abdulaziz University, Jeddah 21589, Saudi Arabia; owais.micro@gmail.com; 4 Karmanos Cancer Institute and Wayne State School of Medicine, Detroit, MI 48201, USA

**Keywords:** catechins, prooxidant, anticancer, copper, DNA breakage, reactive oxygen species, epicatechin, epigallocatechin, epigallocatechin-3-gallate

## Abstract

Catechins, the dietary phytochemicals present in green tea and other beverages, are considered to be potent inducers of apoptosis and cytotoxicity to cancer cells. While it is believed that the antioxidant properties of catechins and related dietary agents may contribute to lowering the risk of cancer induction by impeding oxidative injury to DNA, these properties cannot account for apoptosis induction and chemotherapeutic observations. Catechin (C), epicatechin (EC), epigallocatechin (EGC) and epigallocatechin-3-gallate (EGCG) are the four major constituents of green tea. In this article, using human peripheral lymphocytes and comet assay, we show that C, EC, EGC and EGCG cause cellular DNA breakage and can alternatively switch to a prooxidant action in the presence of transition metals such as copper. The cellular DNA breakage was found to be significantly enhanced in the presence of copper ions. Catechins were found to be effective in providing protection against oxidative stress induced by tertbutylhydroperoxide, as measured by oxidative DNA breakage in lymphocytes. The prooxidant action of catechins involved production of hydroxyl radicals through redox recycling of copper ions. We also determined that catechins, particularly EGCG, inhibit proliferation of breast cancer cell line MDA-MB-231 leading to a prooxidant cell death. Since it is well established that tissue, cellular and serum copper levels are considerably elevated in various malignancies, cancer cells would be more subject to redox cycling between copper ions and catechins to generate reactive oxygen species (ROS) responsible for DNA breakage. Such a copper dependent prooxidant cytotoxic mechanism better explains the anticancer activity and preferential cytotoxicity of dietary phytochemicals against cancer cells.

## 1. Introduction

In recent years, there has been an increasing interest in understanding the potential of cancer chemopreventive properties of plant derived polyphenolic compounds. Epidemiological evidence suggests that a high consumption of dietary products derived from plant sources among certain populations may reduce their risk of cancer induction as compared to those with low intakes [1,2,3]. This has been associated with human food stuff found to be rich in a wide variety of biologically active compounds [4]. Among such dietary constituents, catechins (a major component in green tea) are considered to be the most effective in cancer chemoprevention in humans. Catechins are a sub class of plant polyphenols that particularly include (+)-catechin (C), (−)-epicatechin (EC), (−)-epigallocatechin (EGC), and (−)-epigallocatechin-3-gallate (EGCG) and are considered as the most effective cytotoxic agents and inducers of apoptosis in cancer cells [5,6,7,8]. In recent years, several reports have documented that plant polyphenols including catechins induce apoptosis in various cell lines [9,10,11,12,13]. Of particular interest is the observation that a number of these polyphenols including catechins induce apoptotic cell death in various cell lines but not in normal cells [5,10,11,12,14]. However, the mechanism by which these compounds inhibit cell proliferation and induce apoptosis in cancer cells has been the subject of much interest. These compounds possess both antioxidant as well as prooxidant properties [6,7,15]. Evidence in the literature suggests that the antioxidant properties of such plant polyphenols may not fully account for their observed antiproliferative and cancer therapeutic effects [16]. We earlier proposed a hypothesis where we had explained that the prooxidant, rather than the antioxidant, activity of these compounds is important for their anticancer effects [17,18,19]. Such a prooxidant effect is induced in the presence of transition metals, such as copper. Copper is an important metal ion present in chromatin and is closely associated with DNA bases, particularly guanine [20]. It is one of the most redox active among the various metal ions present in biological systems facilitating rapid recycling, in the presence of molecular oxygen and compounds such as plant polyphenols, leading to the formation of reactive oxygen species (ROS) such as the hydroxyl radical.

**Figure 1:**
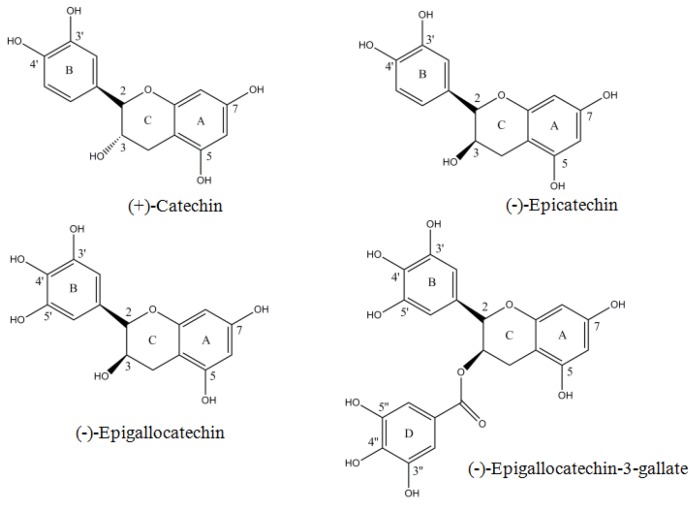
Chemical structure of catechin, epicatechin, epigallocatechin and epigallocatechin-3-gallate.

In this article, we show that catechins can alternately behave as prooxidants in the presence of Cu(II) leading to cytotoxic action. Identification of molecular targets, modulation of which is associated with inhibition of malignantly transformed cells, is vital to cancer prevention and will greatly assist in a better understanding of anticancer mechanisms by naturally occurring chemotherapeutic compounds. A number of reports in the literature have established that tissue, serum, and cellular copper levels are considerably elevated in various malignancies [21,22,23,24]. Therefore, cancer cells may be more subject to electron transfer between copper ions and these catechins to generate ROS [25,26]. Such a mechanism, which involves a copper-dependent pathway of cell death, better explains the anticancer properties of polyphenols of diverse chemical structures, as also their preferential toxicity against cancer cells. The structures of various catechins used in this study are shown in Figure 1.

## 2. Results

### 2.1. Formation of Catechins-Cu(II) Complex

**Figure 2:**
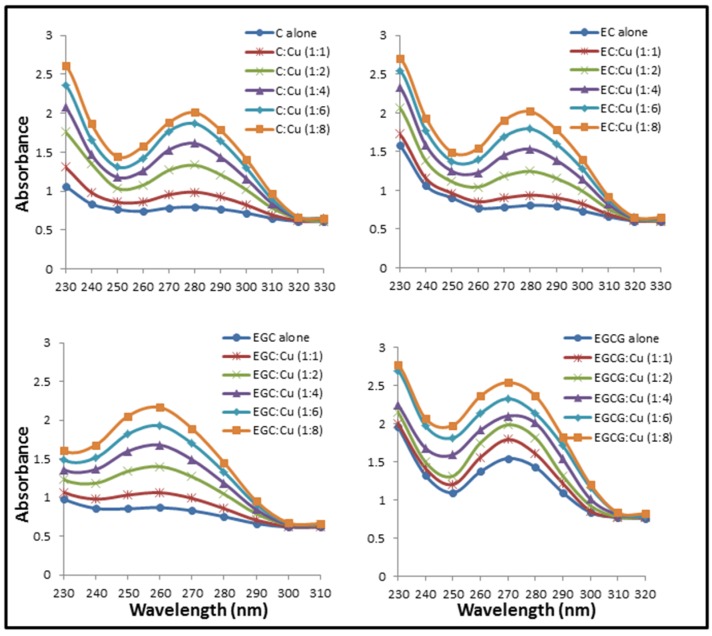
Effect of increasing copper concentrations on the absorbance spectra of Catechin (C), Epicatechin (EC), Epigallocatechin (EGC) and Epigallocatechin-3-gallate (EGCG). Catechins (in 10 mM Tris-HCl, pH 7.5) absorption spectra were recorded in the presence of increasing concentration of Cu(II).

The possibility for the formation of C/EC/EGC/EGCG with Cu(II) complex was examined. This was carried out by recording the absorption spectra of C, EC, EGC and EGCG with increasing concentrations of Cu(II). The results given in Figure 2 show that the addition of Cu(II) to C, EC, EGC and EGCG results in an enhancement in the peak appearing at their respective λ_max_. The absorption spectra of C, EC, EGC and EGCG in the presence of copper suggests a simple mode of interaction between these catechins and Cu(II). The absorption maxima of C, EC, EGC and EGCG lie in the range of 260–280 nm.

### 2.2. Formation of Complexes Involving Cu(II) with Catechins

**Figure 3:**
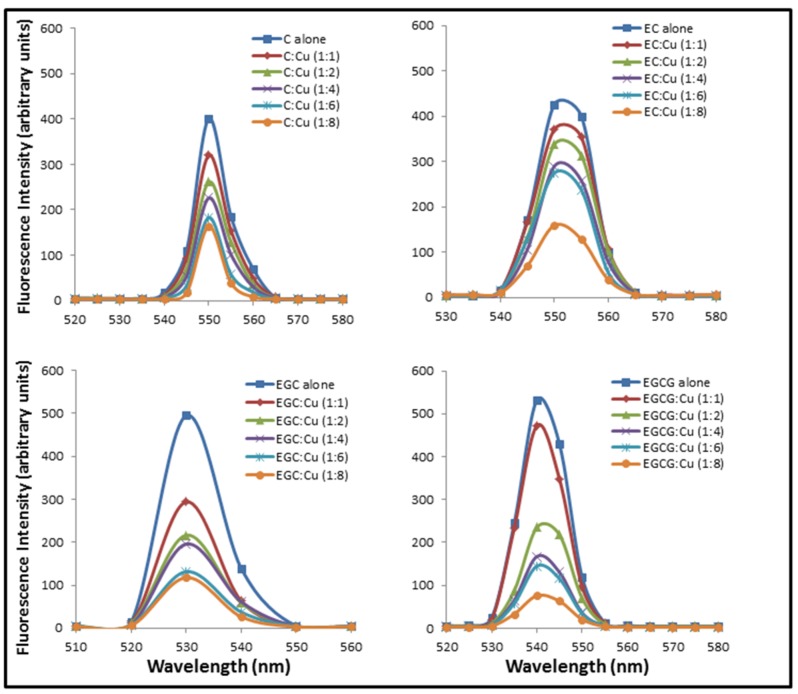
Effect of increasing copper concentrations on the fluorescence emission spectra of C, EC, EGC and EGCG. Catechins (in 10 mM Tris-HCl, pH 7.5) were excited at 273 nm in the presence of increasing concentration of Cu(II) and the emission spectra were recorded between 510 and 580 nm.

Figure 3 shows the effect of addition of increasing molar base pair ratios of Cu(II) on the fluorescence emission spectra of C, EC, EGC and EGCG excited at 273 nm (approximate absorption maximum of catechins). The result shown in Figure 3 indicates binding as addition of Cu(II) causes quenching of C, EC, EGC and EGCG fluorescence. These results support the result of absorption studies shown in Figure 2 where formation of catechins-copper complex was demonstrated.

### 2.3. Detection of Catechins Induced Cu(I) Production by Bathocuproine

**Figure 4:**
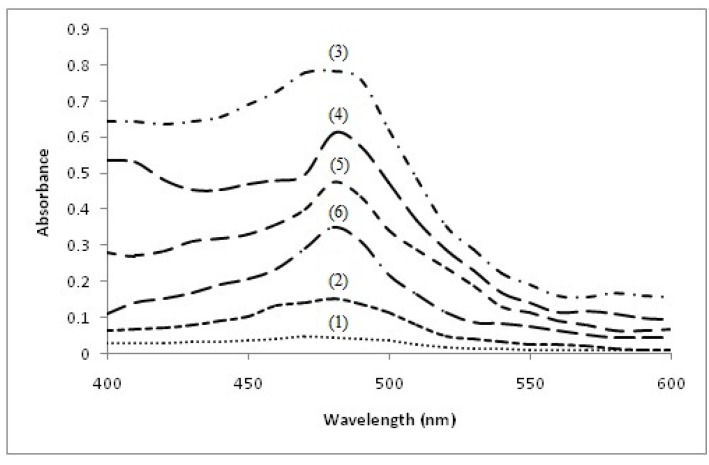
Detection of catechin induced Cu(I) production by Bathocuproine. Reaction mixture (3.0 mL) contained 3.0 mMTris-HCl (pH 7.5) along with 300 µM bathocuproine and indicated concentrations of the following: (1) Bathocuproine + 100 µM Cu(II); (2) Bathocuproine + 100 µM Cu(I); (3) Bathocuproine + 50 µM C + 100 µM Cu(II); (4) Bathocuproine + 50 µM EC + 100 µM Cu(II); (5) Bathocuproine + 50 µM EGC + 100 µM Cu(II); and (6) Bathocuproine + 50 µM EGCG + 100 µM Cu(II). The Bathocuproine alone or Bathocuproine in the presence of respective compounds did not interfere with the Bathocuproine-Cu(I) complex peak at 480 nm (not shown).

The production of Cu(I), formed as a result of reduction of Cu(II) by C, EC, EGC and EGCG, was analyzed using bathocuproine which is a selective Cu(I) sequestering agent that binds specifically to the reduced form of copper, *i.e*., Cu(I), but not to the oxidized form [27]. The Cu(I)-chelates exhibit an absorption maximum at 480 nm. As shown in Figure 4, Cu(II) does not interfere with the maxima, whereas C + Cu(II), EC + Cu(II), EGC + Cu(II) and EGCG + Cu(II) react to generate Cu(I) which complexes with bathocuproine to give a peak appearing at 480 nm. The results show that these catechins are able to reduce Cu(II) to Cu(I) and contribute to the redox cycling of the metal.

### 2.4. Superoxide Production by Catechins

**Figure 5:**
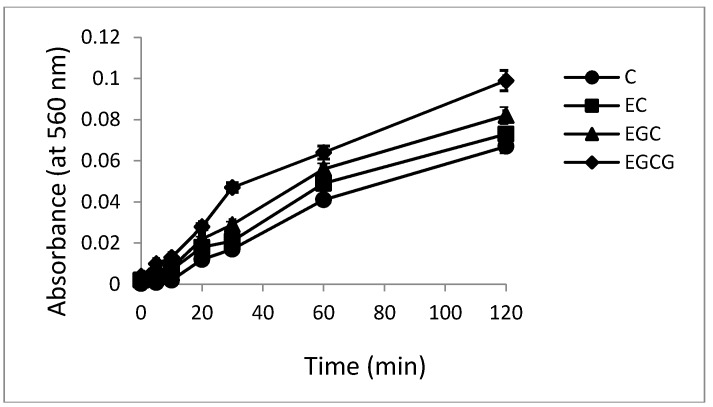
Photogeneration of superoxide anion by catechins on illumination under fluorescent light. Reaction mixture contained 50 mM phosphate buffer (pH 7.5) and 50 μM of C, EC, EGC and EGCG. The samples were placed at a distance of 10 cm from the light source. All values reported are means of three independent experiments. Error bars represent standard error of mean.

The production of superoxide anion was determined by the Nakayama method [28], which involves reduction of NBT by C, EC, EGC and EGCG to a formazan. The time dependent generation of superoxide anion by C, EC, EGC and EGCG as evidenced by the increase in absorbance at 560 nm is shown in Figure 5. The fact that NBT was genuinely assaying superoxide was confirmed by SOD (100 µg/mL) inhibiting the reaction (results not shown). It is known that superoxide may undergo automatic dismutation to form H_2_O_2_ which in the presence of transition metals such as copper favors Fenton type reaction to generate hydroxyl radicals which could act as a proximal DNA cleaving agent leading to oxidative DNA breakage.

### 2.5. Hydroxyl Radical Generation by Catechins

**Figure 6:**
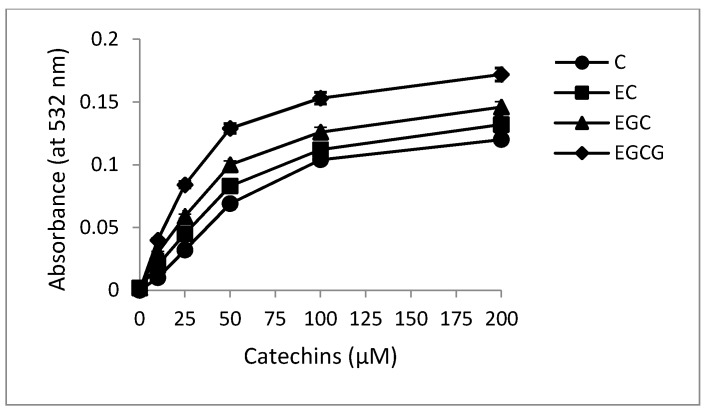
Formation of hydroxyl radicals as a function of catechins concentration in the presence of Cu(II). Reaction mixture (0.5 mL) contained 200 µg calf thymus DNA as substrate, 100 µM Cu(II) and indicated concentrations of C, EC, EGC and EGCG. The reaction mixture was incubated at 37 °C for 1 h. Hydroxyl radical formation was measured by determining the TBA reactive material. All values reported are means of three independent experiments. Error bars represent standard error of mean.

It has been previously shown that during the reduction of Cu(II) to Cu(I), reactive oxygen species such as hydroxyl radicals are formed which serve as the proximal DNA cleaving agent [29]. Therefore, the capacity of C, EC, EGC and EGCG to generate hydroxyl radical in the presence of Cu(II) was examined. The assay is based on the fact that degradation of DNA by hydroxyl radicals results in the release of TBA (2-thiobarbituric acid) reactive material, which forms a colored adduct with TBA whose absorbance is read at 532 nm [30]. The results given in Figure 6 clearly show that increasing concentrations of catechins lead to a progressive increase in the formation of hydroxyl radicals.

### 2.6. Breakage of Calf Thymus DNA by Catechins in the Presence of Cu(II)

C, EC, EGC and EGCG in the presence of Cu(II) were found to generate single strand specific nuclease sensitive sites in calf thymus DNA. The reaction was assessed by recording the proportion of DNA converted to acid soluble-nucleotides by the nuclease. Table 1 gives the dose response data of such a reaction. However, C, EC, EGC and EGCG in the absence of Cu(II) did not show appreciable degradation of calf thymus DNA. Control experiments (data not shown) established that heat denatured DNA underwent 100% hydrolysis following the treatment with nuclease. In the presence of Cu(II) (50 µM), increasing concentrations of C, EC, EGC and EGCG resulted in an increase in nuclease sensitive sites in DNA leading to increased DNA hydrolysis.

**Table 1:** Degradation of calf thymus DNA by the catechins in the presence of Cu(II) as measured by the degree of single strand specific S_1_-nucleasedigestion. Reaction mixture (0.5 mL) containing 10 mM Tris-HCl (pH 7.5) and 500 µg calf thymus DNA was incubated at 37 °C with indicated concentrations of respective polyphenol alone or polyphenol with Cu(II) (100 µM). All values represent mean ± SEM of three independent experiments.

Catechins	Concentration (µM)	% DNA Hydrolyzed	
		without Cu(II)	with Cu(II)
C	50	1.53 ± 0.21	8.87 ± 0.46
	100	3.09 ± 0.37	11.93 ± 0.62
	200	5.98 ± 0.26	17.65 ± 0.73
	300	7.11 ± 0.67	26.34 ± 0.91
EC	50	2.62 ± 0.29	10.43 ± 0.41
	100	4.18 ± 0.35	15.07 ± 0.48
	200	7.26 ± 0.32	22.32 ± 0.81
	300	9.12 ± 0.56	28.56 ± 0.98
EGC	50	3.03 ± 0.43	12.71 ± 0.51
	100	5.21 ± 0.72	16.27 ± 0.81
	200	8.11 ± 0.54	22.45 ± 0.94
	300	10.87 ± 0.76	31.17 ± 1.13
EGCG	50	4.26 ± 0.43	14.11 ± 0.52
	100	8.67 ± 0.59	18.19 ± 0.77
	200	11.89 ± 0.74	29.58 ± 0.83
	300	14.35 ± 0.93	38.74 ± 1.71

### 2.7. Cleavage of Plasmid pBR322 DNA by Catechins

**Figure 7:**
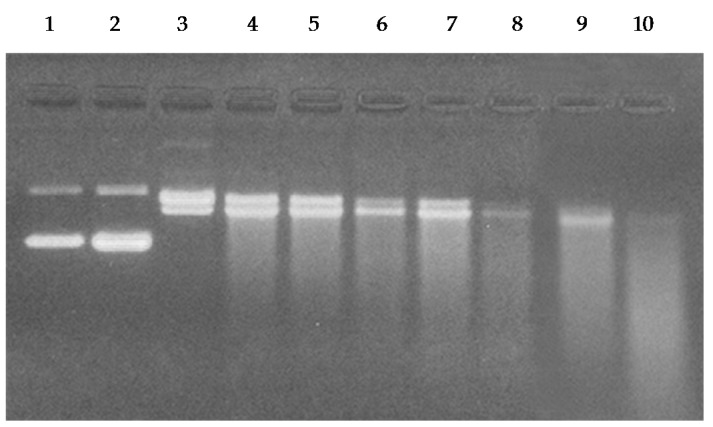
Agarose gel electrophoretic pattern of ethidium bromide stained pBR322 plasmid DNA after treatment with C, EC, EGC and EGCG in the absence and presence of copper. Lane 1: DNA alone; Lane 2: DNA + Cu(II) (50 µM); Lane 3: DNA + C (50 µM); Lane 4: DNA + C (50 µM) + Cu (II) (50 µM); Lane 5: DNA + EC (50 µM); Lane 6: DNA + EC (50 µM) + Cu (II) (50 µM); Lane 7: DNA + EGC (50 µM); Lane 8: DNA + EGC (50 µM) + Cu (II) (50 µM); Lane 9: DNA + EGCG (50 µM); Lane 10: DNA + EGCG (50 µM) + Cu (II) (50 µM).

In order to examine the efficacy of catechins-Cu(II) system in DNA cleavage, as shown in Figure 7, we have tested the ability of C, EC, EGC and EGCG to cause cleavage of supercoiled plasmid pBR322 DNA in the presence of copper ions. As can be seen from the ethidium bromide stained agarose gel pattern, C, EC, EGC and EGCG alone show only some degree of DNA cleavage. However, addition of copper to these four catechins resulted in greater DNA cleavage, demonstrating that catechins are capable of plasmid DNA cleavage in the presence of copper ions.

### 2.8. Cellular DNA Breakage by catechins-Cu(II) in Lymphocytes as Measured by Comet Assay

**Figure 8:**
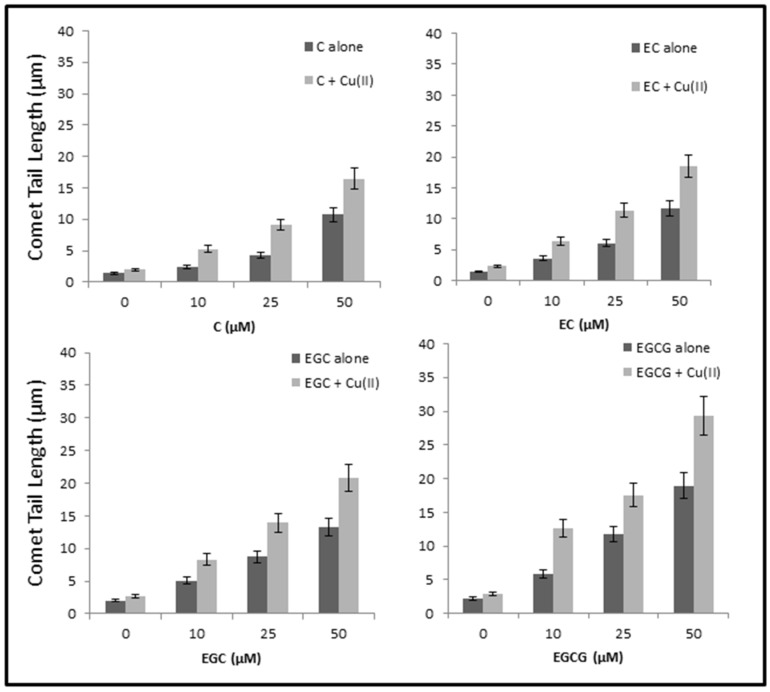
DNA breakage by catechins in human peripheral lymphocytes in the absence and presence of Cu(II). Comet tail length (µms) plotted as a function of increasing concentrations of catechins (0–50 µM) in the absence and presence of 50 µM Cu(II). All points represent mean of three independent experiments. Error bars denote Mean ± SEM. *p* value < 0.05 and significant when compared to control.

We have earlier shown that most of the dietary polyphenolic phytochemicals, which are generally effective antioxidants, can switch to prooxidant action in the presence of transition metals such as copper [17,18]. In the experiment shown in Figure 8, we have tested the ability of C, EC, EGC and EGCG to cause DNA strand breaks in a cellular system of human peripheral lymphocytes both in the absence and the presence of Cu(II), as measured by standard comet assay. As seen from the figure, although all the compounds tested caused some breakage of cellular DNA, the degree of such breakage is enhanced in the presence of copper. Cu(II) (50 µM) controls were similar to untreated lymphocytes without any significant DNA breakage. The results clearly indicate that catechins-Cu(II) system is capable of DNA breakage in isolated lymphocytes and that such cellular DNA breakage is found of the order of EGCG > EGC > EC > C.

### 2.9. Determination of TBARS as a Measure of Oxidative Stress in Nuclei by Catechins in the Presence of Neocuproine and Thiourea

**Figure 9:**
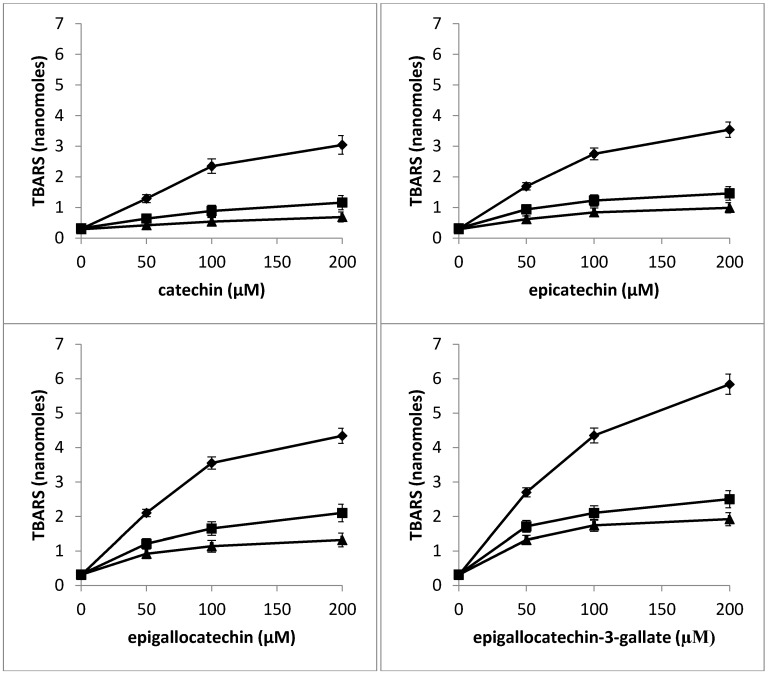
Effect of pre-incubation of lymphocyte nuclei with neocuproine and thiourea on TBARS generated by increasing concentrations of catechins: Catechin alone (filled circle), Catechin + neocuproine (1 mM) (filled square), and Catechin+thiourea (1 mM) (filled triangle). The nuclei suspension was pre-incubated with fixed concentration of neocuproine and thiourea for 30 min at 37 °C, after which it was further incubated for 1 h in the presence of increasing catechins concentration. Values reported are Mean ± SEM of three independent experiments.

According to our hypothesis, the DNA breakage observed in lymphocyte nuclei is the result of the generation of hydroxyl radicals and other reactive oxygen species *in situ*. Oxygen radical damage to deoxyribose or DNA is considered to give rise to TBA reactive material [30,31]. We have therefore determined the formation of TBA reactive substance (TBARS) as a measure of oxidative stress in lymphocyte nuclei with increasing concentrations of C, EC, EGC and EGCG. The effect of pre-incubating the nuclei with neocuproine and thiourea was also studied. Results given in Figure 9 show a dose-dependent increase in the formation of TBA reactive substance in lymphocyte nuclei by C, EC, EGC and EGCG. However, a considerable decrease in the rate of formation of TBARS was observed in the presence of neocuproine and thiourea among all the four catechins used. The results indicate that DNA breakage in nuclei is inhibited by Cu (I) chelation and scavenging of reactive oxygen. Thus, it may be concluded that the oxidative stress induced by polyphenols in lymphocyte nuclei is at least in part mediated by chromatin bound copper.

### 2.10. Antioxidant Activity of Catechins against TBHP-Induced Oxidative Stress in Lymphocytes

**Figure 10:**
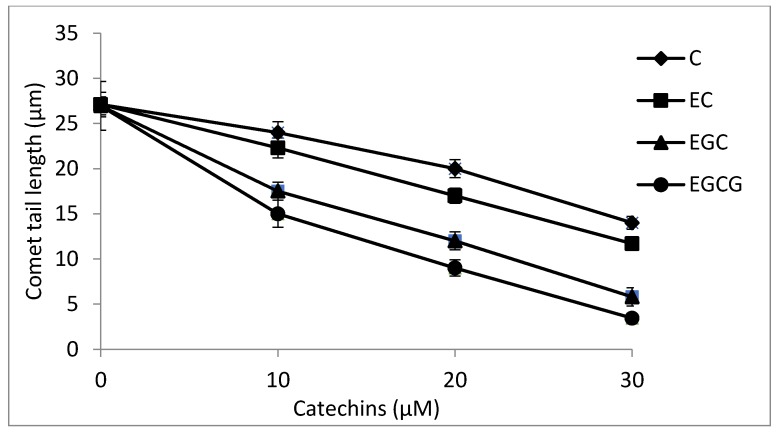
A comparison of antioxidant activities of various catechins as a function of decreasing tail length of comets against TBHP-induced oxidative DNA breakage in human peripheral lymphocytes as assessed by Comet assay. *p* < 0.05 by comparison with TBHP-treated positive control. Values reported are Mean ± SEM of three independent experiments.

TBHP is a well-known inducer of ROS-mediated oxidative stress that results in DNA damage [32,33]. In the present study, we have evaluated the antioxidant potential of C, EC, EGC and EGCG in providing protection to lymphocytes against TBHP induced oxidative injury. Figure 10 shows that whereas all the four catechins were able to inhibit the TBHP-induced lymphocyte DNA degradation, their relative antioxidant activities were different and appeared in the following order: EGCG > EGC > EC > C. The results indicate that EGCG is the most effective antioxidant among the four catechins used.

### 2.11. Catechins Cause Inhibition of Cell Growth in MDA-MB-231 Breast Cancer Cells

**Figure 11:**
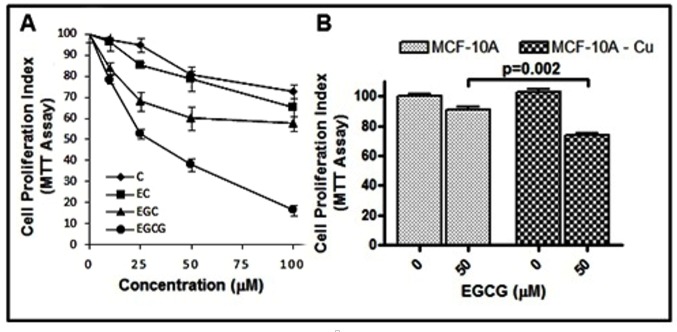
(**A**) The effects of C, EC, EGC and EGCG on the growth of MDA-MB-231 breast cancer cells as detected by MTT assay. The cells were incubated with indicated concentrations of catechins for 48 h, and the results are expressed relative to control (vehicle-treated) cells. (**B**) MCF10A (normal breast epithelial cells) and MCF10A+Cu (MCF-10A cells cultured in copper-enriched medium) were treated with either vehicle (0 μM) or 50 μM EGCG for 72 h.

In Figure 8, it was observed that catechins were able to cause strand breaks in cellular DNA. Subsequently, the effects of the various catechins were tested on the proliferative potential of human breast cancer MDA-MB-231 cells. As can be seen in Figure 11A, a dose-dependent inhibition of proliferation of breast cancer cells MDA-MB-231 by catechins was observed, as assessed by MTT assay. The order of activity was found to be EGCG > EGC > EC > C. These results complement the cellular DNA breakage studies. Further, we observed (Figure 11B) that the normal breast epithelial cells, MCF-10A, were quite resistant to EGCG treatment but their culture in copper-enriched medium resulted in sensitization to EGCG action (*p* < 0.01). These results are in agreement with our earlier published results [5] involving plant polyphenols.

## 3. Discussion

Studies mainly on anticancer mechanisms of plant polyphenols involve the induction of cell cycle arrest and modulation of transcription factors that lead to anti-neoplastic effects [10,34]. In light of the above findings in our laboratory and those of many others in the literature, it may be concluded that the plant polyphenols, particularly present in dietary agents, possessing anticancer and apoptosis-inducing activities are able to mobilize endogenous copper ions, possibly the copper bound to chromatin. Essentially, this would be an alternative, non-enzymatic, and copper-dependent pathway for the cytotoxic action of anticancer agents that are capable of mobilizing and reducing endogenous copper. As such, this would be independent of Fas and mitochondria mediated programmed cell deaths. It is conceivable that such a mechanism may also lead to internucleosomal DNA breakage (a hallmark of apoptosis), as internucleosomal spacer DNA would be relatively more susceptible to cleavage by ROS. Indeed, such a common mechanism better explains the anticancer effects of dietary molecules (*i.e.*, catechins) studied above with diverse chemical structures as also their preferential cytotoxicity toward cancer cells. The generation of hydroxyl radicals in the proximity of DNA is well established as a cause of strand scission. It is generally recognized that such a reaction with DNA is preceded by the association of a ligand with DNA followed by the formation of hydroxyl radicals at that site. The location of the redox-active metal is of utmost importance because the hydroxyl radical, owing to its extreme reactivity, interacts exclusively in the vicinity of the bound metal [35]. Copper ions are known to interact with both DNA phosphates and the bases through coordination binding [36]. Further, copper is also present in chromatin and is closely associated with DNA bases, particularly guanine [20]. Direct interaction of catechins with the DNA bound copper ions in a ternary complex and localized generation of non-diffusible hydroxyl radicals is a likely mechanism involved in catechin/Cu(II)-induced DNA cleavage. It has already been reported by us that the number of galloyl moieties present in catechinsplay an important role in cellular DNA breakage and catechins on reducing copper lead to the formation of “oxidized species” of the compounds. [26]. It may be presumed that the concentration of metals such as copper in cells is a decisive factor in driving antioxidant property of catechins toward their prooxidant action. As already mentioned, cancer cells are known to contain elevated levels of copper [37] and therefore may be more subject to electron transfer with catechins to generate ROS [25,26]. In normal cells, there exists a balance between the free radical generation and the antioxidant defense [38]. However, it has been clearly documented that tumor cells are under persistent oxidative stress and have an altered antioxidant system [39] and thus further ROS stress in malignant cells reaching a threshold level could result in apoptosis [37]. These observations further suggest that neoplastic cells may be more vulnerable to oxidative stress because they function with a heightened basal level of ROS because of increased rate of growth and metabolism [40]. Thus, in cancer cells, an enhanced exposure to ROS generated through the antioxidant/catechin-induced redox activity of endogenous copper can overwhelm the cells antioxidant capacity, leading to irreversible damage and apoptosis.

Beyond the preclinical findings, catechins such as EGCG show little promise as potent chemopreventive agents in the clinical settings, mainly owing to their inefficient systemic delivery and poor bioavailability. Catechins, like other polyphenols, are rapidly metabolized *in vivo*, resulting in a short systemic half-life and low plasma concentrations in the free form. For instance, it has been observed that the peak plasma concentrations reached up to 1.3 µM and 3.1 µM in healthy volunteers receiving 687.5 mg EGCG and 663.5 mg of ECG, respectively [41]. In this context, it needs to be emphasized that the significance of our work lies not so much in the potential therapeutic action of catechins against cancer, but in establishing a principle, namely that it is possible to mobilize elevated levels of endogenous copper in cancer cells by catechins to promote a pro-oxidant cell death. Once such a principle is established, catechins can serve as lead compounds to synthesize/formulate novel, anticancer drugs with superior bioavailability and extended systemic half-life. Presumably, such anticancer drugs would have a better therapeutic impact than the catechins *per se*.

Nevertheless, a previous study from our lab has shown that oral administration of copper to rats can induce a copper overload in their lymphocytes, rendering such isolated lymphocytes more susceptible to EGCG-induced pro-oxidant cellular DNA breakage [42]. This suggests that in cancer cells, where there are considerably higher levels of copper, the concentration of catechins required to elicit such a pro-oxidant cell death mechanism would be significantly lower. Therefore, based on these observations, it is clear that the pro-oxidant action of polyphenols is physiologically feasible provided an appropriate microenvironment with elevated copper levels is present, as is the scenario in cancer cells.

Further, it should be noted that in real life situation, catechins like EGCG are only one of the several polyphenols consumed as part of the diet. Since various other polyphenols present in diet, such as flavonoids and tannins are also active as pro-oxidants [17], their cumulative plasma concentration, bioavailability and anticancer effect should be much greater than a single polyphenol alone. In fact, a combination of EGCG with luteolin, has been found to be more effective than either of the polyphenol alone in inducing apoptosis in cancer cell lines *in vitro* and inhibition of tumor growth in nude mouse xenograft model [43].

## 4. Materials and Methods

### 4.1. Chemicals, Reagents and Cell Lines

(+)-Catechin, (−)-epicatechin, (−)-epigallocatechin, (−)-epigallocatechin-3-gallate, calf thymus DNA, cupric chloride, neocuproine, thiourea, agarose, low melting point agarose, RPMI 1640, Triton X-100, Trypan blue, Histopaque1077, and phosphate buffered saline (PBS) Ca^2+^ and Mg^2+^ free were purchased from Sigma (St. Louis, MO, USA). All other chemicals were of analytical grade. Fresh solutions of C, EC, EGC and EGCG were prepared as a stock of 3.0 mM in double distilled water (ddH_2_O) before use as a stock of 1 mM solution. Upon addition to reaction mixtures, in the presence of buffers mentioned and at concentrations used, all the catechins used remained in solution. The volumes of stock solution added did not lead to any appreciable change in the pH of reaction mixtures. Breast cancer cell line MDA-MB-231 was maintained in DMEM (Invitrogen, Carlsbad, CA, USA) growth media. The medium was supplemented with 10% foetal bovine serum (FBS) and 1% antimycotic antibiotic (Invitrogen, Carlsbad, CA, USA). Normal breast epithelial cells MCF10A were cultured in DMEM/F12 (Invitrogen, Carlsbad, CA, USA) supplemented with 5% horse serum, 20 ng/mL EGF, 0.5 µg/mL hydrocortisone, 0.1 µg/mL cholera toxin, 10 µg/mL insulin, 100 units/mL penicillin and 100 µg/mL streptomycin. Cells were cultured in a 5% CO_2_-humidified atmosphere at 37 °C. MCF10A+Cu cells are MCF10A cells cultured with additional supplementation of 25 µM CuCl_2_ for at least 4 weeks. A 5 mg/mL stock solution of 3-(4,5-dimethylthiazol-2-yl)-2,5-diphenyltetrazolium bromide (MTT) was prepared in PBS.

### 4.2. Absorbance Studies

The effect of increasing concentrations of Cu(II) on absorption spectra of C, EC, EGC and EGCG was observed. The reaction mixture (3.0 mL) contained 10 mMTris-HCl (pH 7.5), 50 µM C, EC, EGC, EGCG and increasing concentrations of Cu(II). The spectra were recorded immediately after addition of all components.

### 4.3. Flourescence Studies

The fluorescence studies were performed on a Shimadzu spectrofluorometer RF-5310 PC (Kyoto, Japan) equipped with a plotter and a calculator. C, EC, EGC and EGCG were excited at their absorption maxima (λ_max_) of 273 nm (approximate absorption maximum of catechins). Emission spectra were recorded in the wavelength range shown in figures.

### 4.4. Detection of Cu(II) Reduction

The selective sequestering agent bathocuproine was employed to detect reduction of Cu(II) to Cu(I) by recording the formation of bathocuproine-Cu(I) complex which absorbs maximally at 480 nm. The reaction mixture (3.0 mL) contained 3.0 mM Tris-HCl (pH 7.5), fixed concentrations (100 µM) of Cu(II) and Cu(I) (for positive control), bathocuproine (300 µM) and of catechins (C, EC, EGC and EGCG) (50 µM). The reaction was started by adding Cu(II) and the spectra were recorded immediately afterwards.

### 4.5. Detection of Superoxide Anion Generation

Superoxide (O_2_^−^) was detected by the reduction of nitroblue tetrazolium (NBT) essentially as described by Nakayama *et al.* [28]. A typical assay mixture contained 50 mM sodium phosphate buffer (pH 7.5), 33 µM NBT, 100 µM EDTA and 0.06% triton X-100 in a total volume of 3.0 mL. The reaction was started by the addition of catechins (C/EC/EGC/EGCG). After mixing, absorbance was recorded at 560 nm at different time intervals, against a blank, which did not contain the compound.

### 4.6. Detection of Hydroxyl Radical Generation

In order to compare the hydroxyl radical production by increasing concentrations of C, EC, EGC and EGCG in the presence of 100 µM Cu(II), the method of Quinlan and Gutteridge [30] was followed. Calf thymus DNA (200 µg) was used as a substrate and the malondialdehyde generated from deoxyribose radicals was assayed by recording the absorbance at 532 nm.

### 4.7. Degradation of Calf Thymus DNA

Single strand specific digestion was performed as described by Wani and Hadi [44]. Reaction mixtures (0.5 mL) contained 10 mM Tris-HCl (pH 7.5), 500 µg of calf thymus DNA and varying amounts of C, EC, EGC, EGCG and cupric chloride (50 µM). All solutions were sterilized before use. Incubation was performed at 37 °C for one hour. The assay determines the acid soluble nucleotides released from DNA as a result of enzyme digestion. Reaction mixture in a total volume of 1.0 mL contained 40 mM Tris-HCl (pH 7.5), 1 mM Magnesium Chloride, water and enzyme. The reaction mixture was incubated at 37 °C for 2 h. The reaction was stopped by adding 0.2 mL bovine serum albumin (10 mg/mL) and 1.0 mL of 14% perchloric acid (chilled). The tubes were immediately transferred to 0 °C for 45 min before centrifugation at 2500 rpm for 10 min at room temperature to remove undigested DNA and precipitated protein. Acid soluble deoxyribonucleotides were determined in the supernatant, colorimetrically, using the diphenylamine method [45]. To a 1.0 mL aliquot, 2.0 mL diphenyl reagent (freshly prepared by dissolving 1 gram of recrystallized diphenylamine in 100 mL glacial acetic acid and 2.75 mL of concentrated H_2_SO_4_) was added. The tubes were heated in a boiling water bath for 30 min. The intensity of blue color was read at 600 nm.

### 4.8. Treatment of pBR322 DNA

Reaction mixture (30 µL) contained 10 mMTris-HCl (pH 7.5), 0.5 µg of plasmid DNA and other components as indicated in legends. Incubation was performed at 37 °C for 2 h. After incubation, 10 µL of solution containing 40 mM EDTA, 0.05% bromophenol blue (tracking dye) and 50% (*v*/*v*) glycerol was added and the solution was subjected to electrophoresis in submarine 1% agarose gel. The gel was stained with ethidium bromide (0.5 µg/mL), viewed and photographed on a UV-transilluminator.

### 4.9. Isolation of Lymphocytes

Heparinized blood samples (2 mL) from a single, healthy, non-smoking donor was obtained by venepuncture and diluted suitably in Ca^2+^ and Mg^2+^ free PBS. Lymphocytes were isolated from blood using Histopaque 1077 (Sigma Diagnostics, St Louis, MS, USA), and the cells were finally suspended in RPMI 1640.

### 4.10. Viability Assessment of Lymphocytes

The lymphocytes were checked for their viability before the start and after the end of the reaction using Trypan Blue Exclusion Test by Pool-Zobel *et al*. [46]. The viability of the cells was found to be greater than 93%.

### 4.11. Comet Assay

Comet assay was performed under alkaline conditions essentially according to the procedure of Singh *et al.* [47] with slight modifications. Fully frosted microscopic slides precoated with 1.0% normal melting agarose at about 50 °C (dissolved in Ca^2+^ and Mg^2+^ free PBS) were used. Approximately 10,000 cells were mixed with 75 µL of 2.0% LMPA to form a cell suspension and pipetted over the first layer and covered immediately by a coverslip. The agarose layer was allowed to solidify by placing the slides on a flat tray and keeping it on ice for 10 min. The coverslips were removed and a third layer of 0.5% LMPA (75 µL) was pipetted and coverslips placed over it and kept on ice for 5 min for proper solidification of layer. The coverslips were removed and the slides were immersed in cold lysing solution containing 2.5 M NaCl, 100 mM EDTA, 10 mM Tris, pH 10, and 1% Triton X-100 added just prior to use for a minimum of 1 h at 4 °C. After lysis DNA was allowed to unwind for 30 min in alkaline electrophoretic solution consisting of 300 mM NaOH, 1 mM EDTA, pH > 13. Electrophoresis was performed at 4 °C in a field strength of 0.7 V/cm and 300 mA current. The slides were then neutralized with cold 0.4 M Tris, pH 7.5, stained with 75 µL Ethidium Bromide (20 µg/mL) and covered with a coverslip. The slides were placed in a humidified chamber to prevent drying of the gel and analyzed the same day. Slides were scored using an image analysis system (Komet 5.5, Kinetic Imaging, Liverpool, UK) attached to a Olympus (CX41) fluorescent microscope and a COHU 4910 (equipped with a 510–560 nm excitation and 590 nm barrier filters) integrated CC camera. Comets were scored at 100× magnification. Images from 50 cells (25 from each replicate slide) were analyzed. The parameter taken to assess lymphocytes DNA damage was tail length (migration of DNA from the nucleus, µm) and was automatically generated by Komet 5.5 image analysis system.

Treatment of intact lymphocytes with four catechins (C, EC, EGC and EGCG) and the subsequent Comet assay was performed essentially as described earlier by Azmi *et al*. [48]. For antioxidant study [49], the cells were preincubated with polyphenols in eppendorf tubes in a reaction volume of 1.0 mL. After the preincubation (for 30 min at 37 °C), the reaction mixture was centrifuged at 4000 rpm, the supernatant was discarded and the pelleted lymphocytes were resuspended in 100 µL of PBS (Ca^2+^ and Mg^2+^ free) and layered for further treatment with TBHP (50 µM). The incubation period was 30 min at 37 °C in dark. The other conditions remained the same as described above.

### 4.12. Determination of TBARS

Thiobarbituric acid reactive substance was determined according to the method of Ramanathan *et al.* [50]. A cell suspension (1 × 10^5^/mL) was incubated with C, EC, EGC and EGCG (0–200 µM) at 37 °C for 1 h and then centrifuged at 1000 rpm. In some experiments the cells were pre-incubated with fixed concentrations of neocuproine and thiourea. The cell pellet was washed twice with phosphate buffered saline (Ca^2+^ and Mg^2+^ free) and suspended in 0.1 N NaOH. This cell suspension (1.4 mL) was further treated with 10% TCA and 0.6 M TBA (2-thiobarbituric acid) in boiling water bath for 10 min. The absorbance was read at 532 nm and converted into nmoles of TBA reactive substance using the molar extinction coefficient (1.56 × 9 × 105 M^−1^·cm^−1^).

### 4.13. Cell Growth Inhibition Studies by MTT Assay

MDA-MB-231 cells were seeded at a density of 1 × 10^4^ cells per well in 96-well microtiter culture plates. After overnight incubation, normal growth medium was removed and replaced with either fresh medium (untreated control) or different concentrations of respective catechins in growth medium. After the desired time of incubation (48 h), MTT solution was added to each well (0.1 mg/mL in DMEM) and incubated further for 4 hours at 37 °C. Upon termination, the supernatant was aspirated and the MTT formazan, formed by metabolically viable cells, was dissolved in a solubilisation solution containing DMSO (100 µL) by mixing for 5 min on a gyratory shaker. The absorbance was measured at 540 nm (reference wavelength 690 nm) on an Ultra Multifunctional Microplate Reader (Bio-Rad, Hercules, CA, USA). Absorbance of control (without treatment) was considered as 100% cell survival. Each treatment had four replicate wells and the mean values were plotted.

### 4.14. Statistics

The statistical analysis was performed as described by Tice *et al*. [51] and is expressed as mean ± SEM/SD of three independent experiments. A student’s *t*-test was used for examining statistically significant differences. Analysis of variance was performed using ANOVA. *p* values < 0.05 were considered statistically significant.
